# Chaihu-Wendan Decoction alleviates obesity through PTEN-mediated uncoupling of metabolic signaling and macrophage activation

**DOI:** 10.3389/fendo.2026.1779657

**Published:** 2026-04-15

**Authors:** Bin Ye, Yujie Zhou, Yanling Wang, Qian Shi, Siqin Huang, Haiyan Yang, Baoxin Mao, Ping Wang

**Affiliations:** 1 Graduate School of Jiangxi University of Chinese Medicine, Nanchang, Jiangxi, China; 2 College of Integrated Traditional Chinese and Western Medicine, Nanchang Medical College, Nanchang, Jiangxi, China

**Keywords:** Chaihu Wendan Decoction, immune microenvironment, macrophage phenotype switching, metabolic inflammation, PTEN/AKT/mTORsignaling pathway

## Abstract

**Objective:**

Obesity is increasingly recognized as an immunometabolic disorder driven by dysregulated crosstalk between visceral adipose tissue and the liver, particularly along the liver-omentum axis, which promotes insulin resistance and hepatic steatosis. Although Chaihu-Wendan Decoction (CHWD) is effective for metabolic disorders, its molecular mechanisms of action on this inflammatory axis remains unclear. This study aimed to investigate the therapeutic mechanisms of CHWD in high-fat diet (HFD)-induced obesity model, specifically focusing on insulin signaling and immune microenvironment remodeling in the liver-omentum axis.

**Method:**

C57BL/6J mice were fed a HFD to induce obesity and treated with CHWD. Metabolic phenotypes were assessed via biochemical and histological analyses. The molecular mechanisms were explored by evaluating the PTEN/PI3K/AKT/mTOR pathway and omental macrophage profiles using Western blot, ELISA, and immunohistochemistry.

**Results:**

CHWD treatment significantly ameliorated HFD-induced body weight gain, dyslipidemia, and hepatic steatosis. Mechanistically, CHWD acted as a regulator of PTEN-associated signaling, which triggered a dual-regulation of PTEN/AKT/mTOR signaling, i.e., robust reactivation of upstream insulin signaling (INSR/IRS1/PI3K/AKT) coupled with the paradoxical suppression of downstream mTOR phosphorylation. This “uncoupling” process restored insulin sensitivity without promoting lipogenesis. Concurrently, CHWD remodeled the omental immune microenvironment by restoring omentin-1 secretion and promoting macrophage phenotype switching, characterized by maintenance of a CD68^+^ macrophage population accompanied by suppression of iNOS-mediated cytotoxic effector functions.

**Conclusion:**

CHWD alleviates HFD-induced obesity and metabolic inflammation by coordinately targeting the PTEN/AKT/mTOR axis and reprogramming omental immunity. These findings provide the primary evidence supporting that CHWD modulates the liver-omentum axis via distinct signaling and immune mechanisms, offering a novel therapeutic strategy for metabolic syndrome.

## Highlights

- CHWD uncouples AKT from mTOR via PTEN to boost insulin sensitivity/cut lipids.
- Phospho-homeostasis reprograms macrophages to a homeostatic rather than M1 state.
- The liver-omentum axis conveys Omentin-1 to target hepatic steatosis via portal flow.

## 1 Introduction

Obesity is a major global health challenge, with its prevalence having risen sharply over the past decades. It is a key driver of metabolic syndrome, type 2 diabetes, and non-alcoholic fatty liver disease (1). As reported by the World Health Organization, the total number of adults worldwide suffering from excess weight is about 2.5 billion, with more than 890 million suffering from obesity. Beyond excess adiposity, obesity is now recognized as a chronic inflammatory condition in which visceral adipose tissue serves as a primary source of metabolic and immune dysregulation (2). In this state, adipose-derived inflammatory signals impair insulin action, disturb lipid handling, and contribute to the progression of hepatic steatosis (3, 4). Although current pharmacotherapies such as GLP-1 receptor agonists have improved clinical outcomes, their high costs, tolerability issues, and limited long-term accessibility underscore the need for alternative strategies that are both effective and sustainable (5).

The pathophysiology of metabolic dysfunction in obesity is an intricate process involving the coordination of multiple organs. Recent studies identify the greater omentum as an active immunometabolic organ that serves as a critical regulatory node in obesity-associated metabolic inflammation (6). The greater omentum is anatomically linked to the liver via portal venous drainage, functioning as an endocrine and immune reservoir situated upstream of the hepatic blood supply. Consequently, it directly influences liver physiology through the release of adipokines and inflammatory mediators into the portal vein (7, 8). Obesity disrupts this communication as follows: beneficial adipokines, such as omentin-1, decline, while macrophage inflammatory activity increases body weight exceeded 20%, creating an adverse portal microenvironment that aggravates hepatic steatosis and systemic insulin resistance (9, 10). This functional “liver-omentum axis” offers a new conceptual framework for understanding metabolic inflammation and presents an attractive therapeutic target (11).

Traditional Chinese Medicine (TCM) has long emphasized the interconnectedness of the liver and abdominal membranes in disorders characterized by “Phlegm-Dampness” and impaired metabolic fluid dynamics (12). Chaihu-Wendan Decoction (CHWD), a classic Traditional Chinese Medicine (TCM) formula, has been employed for centuries to treat disorders associated with “Phlegm-Dampness” and “Gallbladder-Stomach disharmony”—concepts that align with modern metabolic and digestive disturbances (13). It is widely applied in clinical practice for obesity-related conditions, yet its mechanistic basis remains insufficiently defined within modern biological models (14, 15). CHWD excels at clearing pathogens lodged between internal organs, suggesting a potential specificity for omental intervention. However, whether CHWD can reconfigure the omental immune landscape and modulate the PTEN-mediated signaling cascade remains unexplored.

In the present study, we investigated the therapeutic effects and underlying mechanisms of CHWD in a high-fat diet-induced obesity model, with particular attention to the liver–omentum axis. We examined whether CHWD could improve metabolic dysfunction by restoring omentin-1 secretion, alleviating omental inflammation, and modulating hepatic insulin-related signaling pathways. By integrating metabolic, inflammatory, histological, and tissue signaling analyses, this study aims to clarify the mechanistic basis of CHWD in obesity-associated metabolic dysfunction and to evaluate the liver–omentum axis as a potential target of intervention.

## 2 Materials and methods

### 2.1 Materials

Preparation of Chaihu Wendan Decoction: The Chaihu-Wendan Decoction (CHWD) used in this study is a classic herbal formula composed of eight crude medicinal materials: Chaihu [the root of *Bupleurum chinense* DC. (12 g)], Huangqin [the root of *Scutellaria baicalensis* Georgi (9 g)], Banxia [the rhizome of *Pinellia ternata* (Thunb.) Makino (9 g)], Chenpi [the pericarp of Citrus *reticulata* Blanco (9 g)], Fuling [the sclerotium of *Wolfiporia cocos* (F.A.Wolf) Ryvarden & Gilb. (12 g)], Zhishi [the immature fruit of *Citrus × aurantium* L. (9 g)], Zhuru [the stem of *Phyllostachys edulis* (Carrière) J.Houz. (9 g)], and Gancao [the root of *Glycyrrhiza uralensis* Fisch. ex DC. (6 g)]. All botanical names were taxonomically validated according to the Plants of the World Online (POWO) database. The mixed herbal materials were decocted three times with 30-fold distilled water (w/w) at 100 °C for 30 min each time. The resulting extracts were combined, filtered, and concentrated to a final volume of 20 mL using a rotary evaporator under reduced pressure.

ELISA Kits and Biochemical Assays: The levels of murine IFN-γ, IL-6, iNOS, LPS, TNF-α, and Omentin-1 were determined using Enzyme-linked immunosorbent assay (ELISA) kits from Jiangsu Meimian Industrial Co., Ltd. (Jiangsu, China). Liver and kidney function parameters were measured using assay kits provided by Shenzhen LWPOCT (Shenzhen, China).

Antibodies and Kits: Primary antibodies against STAT3 (AF6294, 1:1000), HIF1A (AF1009, 1:1000), mTOR (AF6308, 1:1000), p-mTOR (AF3308, 1:1000), BCL2 (AF6139, 1:1000), p-IRS1 (AF3272, 1:1000), p-PI3K (AF3242, 1:1000), and PTEN (AF5447, 1:1000) were purchased from Affinity Biosciences (Cincinnati, OH, USA). Primary antibodies targeting AKT (10176-2-AP, 1:2000), p-AKT (66444-1-Ig, 1:2000), p-INSR (31133-1-AP, 1:1000), and β-actin (81115-1-RR, 1:5000), along with HRP-conjugated secondary antibodies (Goat anti-Rabbit, SA00001-2; Goat anti-Mouse, SA00001-1; 1:5000), were obtained from Proteintech Group (Rosemont, IL, USA/Wuhan, China). Additionally, Goat anti-Rabbit IgG-HRP (Cat# CSE134) was sourced from Shanghai Kureis Biotech (Shanghai, China).

### 2.2 Animals

A total of 142 specific pathogen-free (SPF) male C57BL/6J mice (4 weeks old, 12–14 g) were purchased from Hunan SJA Laboratory Animal Co., Ltd. (Changsha, China) [License No. SCXK (Xiang) 2021-0002; Certificate No. 430727241102910225]. Mice were housed in the SPF facility of Jiangxi University of Chinese Medicine under controlled environmental conditions (25 ± 2 °C, 60–70% relative humidity, 12-h light/dark cycle) with *ad libitum* access to food and water. All experimental procedures complied with the “3R” principles and the *Guide for the Care and Use of Laboratory Animals*. The study protocol was approved by the Experimental Animal Ethics Committee of Jiangxi University of Chinese Medicine (Approval No. JZLLSC20250524).

### 2.3 Establishment of the animal model and experimental design

After one week of acclimatization, mice were randomly assigned to a normal control group (n = 12) or a model group (n = 130). The normal control group was maintained on a standard chow diet, while the model group was fed a high-fat diet (HFD, 60% kcal from fat; Research Diets) for 8 weeks to induce obesity. After 8 weeks, the body weight of the mice was measured. The obesity model was considered successfully established when the body weight of mice in the modeling group exceeded the average weight of the normal group by 20% (16).

Top 60 mice meeting the obesity criteria were selected from the model group and randomized into five subgroups (n = 12 per group). The study comprised six groups in total: 1) Normal Control Group (Control): Standard diet + vehicle (purified water). 2) Model Control Group (Model): HFD-induced + vehicle. 3) Positive Drug Control Group (PC): Orlistat (27.3 mg/kg/day). 4) CHWD Low-Dose Group (CHWD-L): 9.75 g/kg/day.5) CHWD Medium-Dose Group (CHWD-M): 19.50 g/kg/day. 6) CHWD High-Dose Group (CHWD-H): 39 g/kg/day. Intervention Protocol: From week 9 to week 12, all groups (including the Model and treatment groups) were switched to a standard basal feed to mimic a dietary intervention scenario. Treatments were administered via intragastric gavage once daily for 4 consecutive weeks. Dosages were calculated based on the human-mouse body surface area conversion.

### 2.4 Omental tissue sampling

Mice were sacrificed by cervical dislocation and secured in a supine position on a dissection board. The abdominal skin was sterilized with 75% ethanol. A midline incision was made using sterile surgical scissors, extending from the pubic symphysis to the xiphoid process. The skin and abdominal muscle layers were carefully opened along the linea alba, with care taken to avoid damaging blood vessels and contaminating the tissue. The greater omentum was identified as a translucent, apron-like adipose structure covering the small intestine, attached to the greater curvature of the stomach and the transverse colon. The free edge of the omentum was gently lifted using sterile forceps, and the tissue was surgically dissected from its attachments. The excised omentum was immediately immersed in ice-cold sterile PBS and rinsed 2–3 times to remove residual blood and debris. Depending on experimental requirements, the tissue was either snap-frozen in liquid nitrogen for storage or processed immediately for downstream analysis. Carcasses were disposed of in accordance with institutional biosafety guidelines (17).

### 2.5 Biochemical analysis and ELISA

At the end of the experiment, blood samples were collected and centrifuged at 4 °C to obtain serum. Liver and kidney function indices (AST, ALT, TG, TC, HDL-C and LDL-C) and glucose (GLU) were quantified using an automatic biochemical analyzer (LWPOCT, PBC22A Plus, Shenzhen, China). Serum concentrations of inflammatory cytokines (TNF-α, IL-6, IFN-γ), LPS, iNOS, and Omentin-1 were determined using commercial ELISA kits following the manufacturer’s protocols. Optical density (OD) values were measured using a multimode microplate reader (18).

### 2.6 Histopathology and immunohistochemistry

Liver and greater omental tissues were excised, fixed in 4% paraformaldehyde, and embedded in paraffin. Tissue sections (4-5 μm thick) were obtained using a rotary microtome (Leica Microsystems, Germany). Following deparaffinization and rehydration, sections were stained with Hematoxylin and Eosin (H&E) for histopathological examination. The experimental procedures were performed as previously described (19).

### 2.7 Western blot analysis

Tissue samples were homogenized in RIPA lysis buffer containing protease and phosphatase inhibitors using a high-speed tissue grinder. Protein concentration was determined using the BCA assay. Equal amounts of protein (30-50 μg) were separated by SDS-PAGE and transferred onto PVDF membranes. Membranes were blocked with QuickBlock™ buffer and incubated overnight at 4 °C with specific primary antibodies against STAT3, HIF1A, AKT, p-AKT, mTOR, p-mTOR, BCL2, p-INSR, p-IRS1, p-PI3K, PTEN, and β-actin. Following incubation with HRP-conjugated secondary antibodies, protein bands were visualized using an ECL chemiluminescence kit and captured with a chemiluminescence imaging system (20).

### 2.8 Immunofluorescence of CD68 and iNOS in whole-mount omental tissue

The greater omentum was surgically resected and washed in ice-cold PBS to remove blood contaminants. The whole tissues were spread flat and fixed in 4% paraformaldehyde (PFA) for 2–4 hours at 4°C. After washing three times with PBS, the tissues were permeabilized and blocked in PBS containing 0.5% Triton X-100 and 5% bovine serum albumin (BSA) for 1 hour at room temperature. Samples were then incubated overnight at 4 °C with primary antibodies purchased from Abcam (Cambridge, UK): rat anti-mouse CD68 (1:200, ab53444) for total macrophage detection and rabbit anti-mouse iNOS (1:100, ab15323) to identify M1-polarized macrophages (21).

Following primary antibody incubation, the tissues were washed with PBS and incubated for 1 hour at room temperature in the dark with species-specific fluorophore-conjugated secondary antibodies: Goat Anti-Rat IgG H&L (Alexa Fluor^®^ 594, ab150160, Abcam) and Goat Anti-Rabbit IgG H&L (Alexa Fluor^®^ 488, ab150077, Abcam). Nuclei were counterstained with DAPI. Fluorescence images were acquired using a fluorescence microscope (DMi8, Leica Microsystems). Finally, the iNOS^+^ and CD68^+^ cells were quantified using ImageJ software.

### 2.9 Statistical analysis

Statistical analyses were performed using GraphPad Prism 10.0 software. Data are expressed as mean ± standard deviation (SD). Differences among multiple groups were analyzed using one-way analysis of variance (ANOVA), followed by the Tukey’s multiple comparisons test for *post hoc* pairwise comparisons. A *P*-value < 0.05 was considered statistically significant.

## 3 Results

### 3.1 Validation of the high-fat diet-induced obesity mouse model

**Figure 1:**
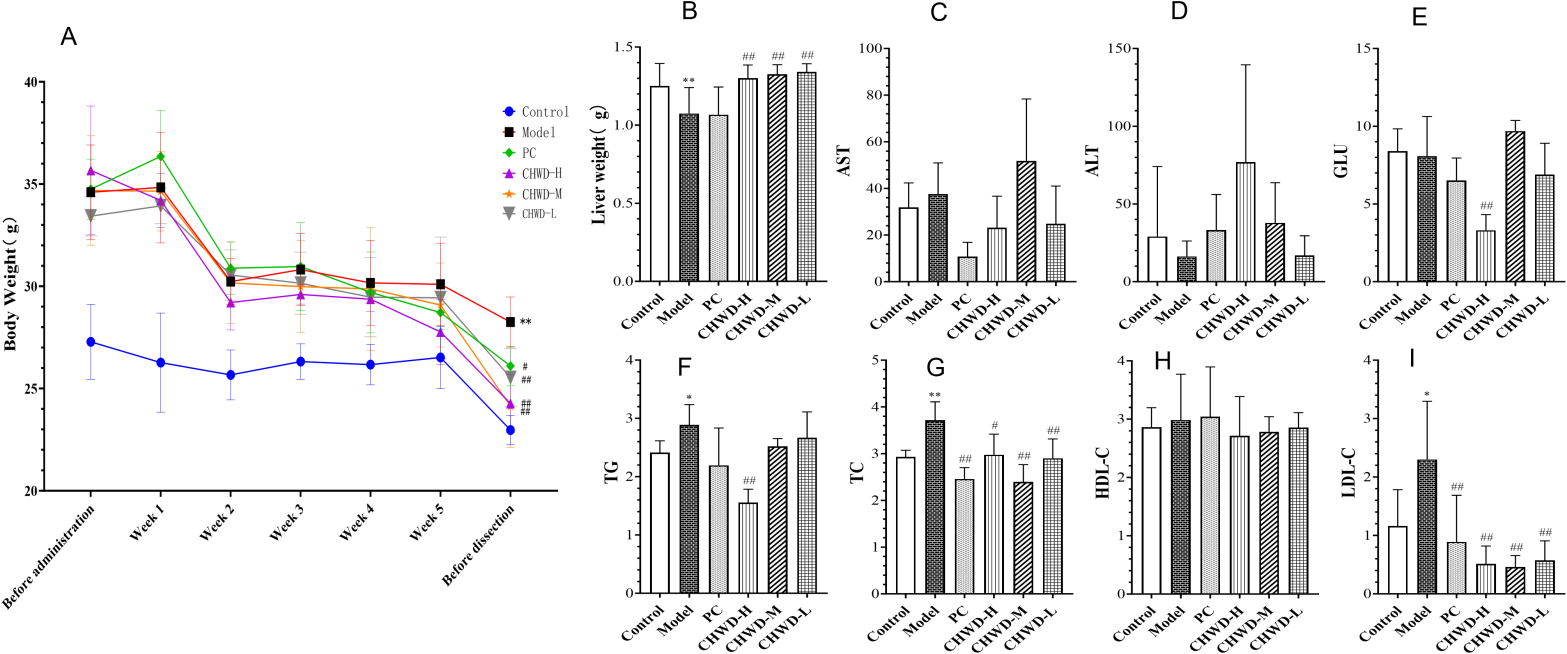
Effects of Chaihu-Wendan Decoction (CHWD) on metabolic parameters in HFD-induced obese mice. **(A)** Body weight. **(B)** Liver weight. **(C)** Level of AST. **(D)** Level of ALT. **(E)** Level of GLU. **(F)** Level of TG. **(G)** Level of TC. **(H)** Level of HDL-C. **(I)** Level of LDL-C. One asterisk (*) indicates a difference, while two asterisks (**) indicate a significant difference. One hash (#) indicates a difference (p < 0.05), and two hashes (##) indicate a significant difference (p < 0.01).

**Figure 2:**
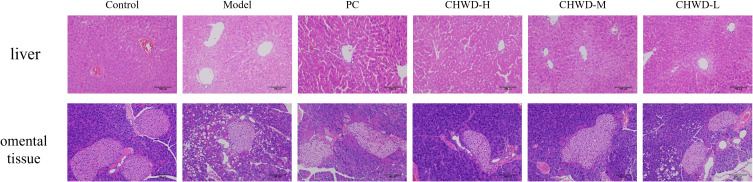
Representative H&E-stained photomicrographs of liver and greater omental tissues across experimental groups. (magnification of ×200).

**Figure 3:**
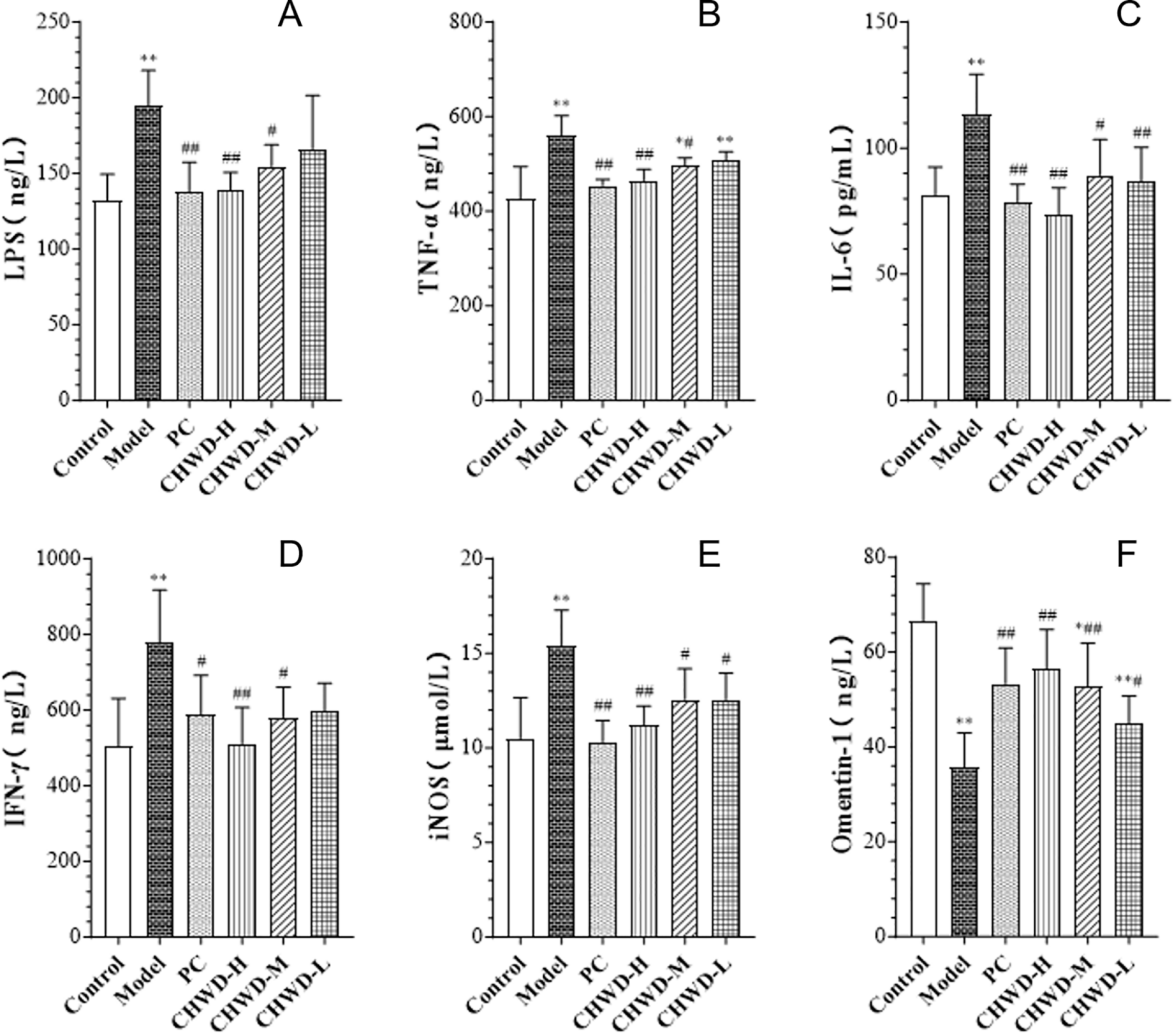
Effects of CHWD on systemic inflammatory mediators, including LPS, TNF-α, IFN-γ, and iNOS, and serum omentin-1 levels. **(A)** LPS. **(B)** TNF. **(C)** IL-6. **(D)** IFN-γ. **(E)** iNOS. **(F)** Omentin-1. Data are expressed as mean ± SD (n = 6). The significant differences are based on P < 0.05 (*) and P < 0.01 (**) vs. Control group and based on P < 0.05 (#) and P < 0.01 (##) vs. Model group.

To establish the experimental foundation for this study, we first validated the obesity mouse model induced by an 8-weeks feeding of HFD (**Figure 1**). Mice in the model group exhibited a significant increase in both body weight and liver weight compared to the Normal Control group, indicating successful induction of the obese phenotype. Histopathological analysis of liver tissue (**Figure 2**) further confirmed these macroscopic findings. The Control group displayed an intact hepatic lobular architecture with orderly arranged hepatic cords and centrally located nuclei, showing no signs of lipid accumulation. In contrast, the model group presented severe hepatic steatosis, characterized by the diffuse presence of macro- and micro-vesicular lipid vacuoles within the cytoplasm, distinct ballooning degeneration of hepatocytes, and disarray of hepatic cords. Consistent with these pathological alterations, the model group exhibited a significant elevation in serum inflammatory markers, including TNF-α, iNOS, IL-6, and IFN-γ, compared to the Control group (**Figure 3**). Collectively, these phenotypic, histopathological, and biochemical changes confirmed the successful establishment of the HFD-induced obesity model.

Quantitative analysis is performed on general physical characteristics and serum biochemical indices. Data are presented as mean ± SD (n = 12). Statistical significance is determined by one-way ANOVA based on *P* < 0.05 (*) and *P* < 0.01 (**) vs. Normal Control group and based on *P* < 0.05 (#) ad *P* < 0.01 (##) vs. Model group.

### 3.2 Chaihu-Wendan Decoction ameliorates HFD-induced obesity, dyslipidemia, and hepatic steatosis

To evaluate the therapeutic efficacy of CHWD, we established a diet-induced obesity (DIO) mouse model. As expected, mice fed a high-fat diet (HFD) for 8 weeks exhibited significantly increased body weight and liver weight compared to the Normal Control group, confirming the successful establishment of the model (**Figure 1**). Following 4 weeks of intervention, CHWD treatment effectively reversed these phenotypic alterations.

Serum biochemical analysis revealed that the model group suffered from severe dyslipidemia. CHWD treatment significantly reduced the levels of Triglycerides (TG), Total Cholesterol (TC), and Low-Density Lipoprotein Cholesterol (LDL-C). Notably, the High-Dose group (CHWD-H) exhibited the most profound lipid-lowering effect, significantly reducing all three parameters (TG, TC, LDL-C) compared to the model group, while the Medium- (CHWD-M) and Low-Dose (CHWD-L) groups also significantly lowered TC and LDL-C levels (**Figure 1**).

Histological assessment (H&E staining) further confirmed these findings. In the liver, the model group displayed severe hepatic steatosis, characterized by diffuse macro- and micro-vesicular lipid vacuoles, ballooning degeneration of hepatocytes, and disorganized hepatic cords. CHWD intervention, particularly at the high dose, markedly alleviated lipid accumulation and partially restored hepatic lobular architecture.

Similarly, in the greater omental adipose tissue, the model group exhibited marked adipocyte hypertrophy, with enlarged unilocular lipid droplets occupying nearly the entire cytoplasmic space. Adipocytes displayed irregular cell boundaries and focal disruption of membrane integrity. In addition, prominent infiltration of inflammatory cells was observed in the inter-adipocyte spaces, forming scattered crown-like structures surrounding hypertrophic adipocytes. The stromal-vascular compartment appeared expanded and loosely organized, suggesting pathological remodeling of visceral adipose tissue under high-fat diet conditions.

In contrast, CHWD treatment reduced adipocyte size, attenuated inflammatory cell infiltration, and improved overall tissue organization, restoring a more uniform and compact adipose architecture (**Figure 2**).

### 3.3 CHWD mitigates systemic inflammation and restores the secretion of the beneficial adipokine omentin-1

Chronic low-grade visceral inflammation is a hallmark of obesity. The results showed that HFD feeding triggered a systemic inflammatory response, indicated by significantly elevated serum levels of LPS, TNF-α, IL-6, IFN-γ, and iNOS compared to the Control group. Conversely, the serum level of omentin-1, an anti-inflammatory and insulin-sensitizing adipokine, was significantly suppressed in the model group.

CHWD treatment potently reversed this pro-inflammatory profile. The CHWD-H and CHWD-M groups showed significantly lower levels of LPS, TNF-α, IL-6, and IFN-γ compared to the model group. Furthermore, CHWD treatment (at all doses) significantly inhibited iNOS production and, crucially, restored serum omentin-1 levels (**Figure 3**). These results suggest that CHWD attenuated systemic inflammatory responses and repaired the endocrine dysfunction of the adipose tissue.

### 3.4 CHWD restores insulin sensitivity via the PTEN/AKT signaling while uncoupling mTOR-mediated lipogenesis

**Figure 4:**
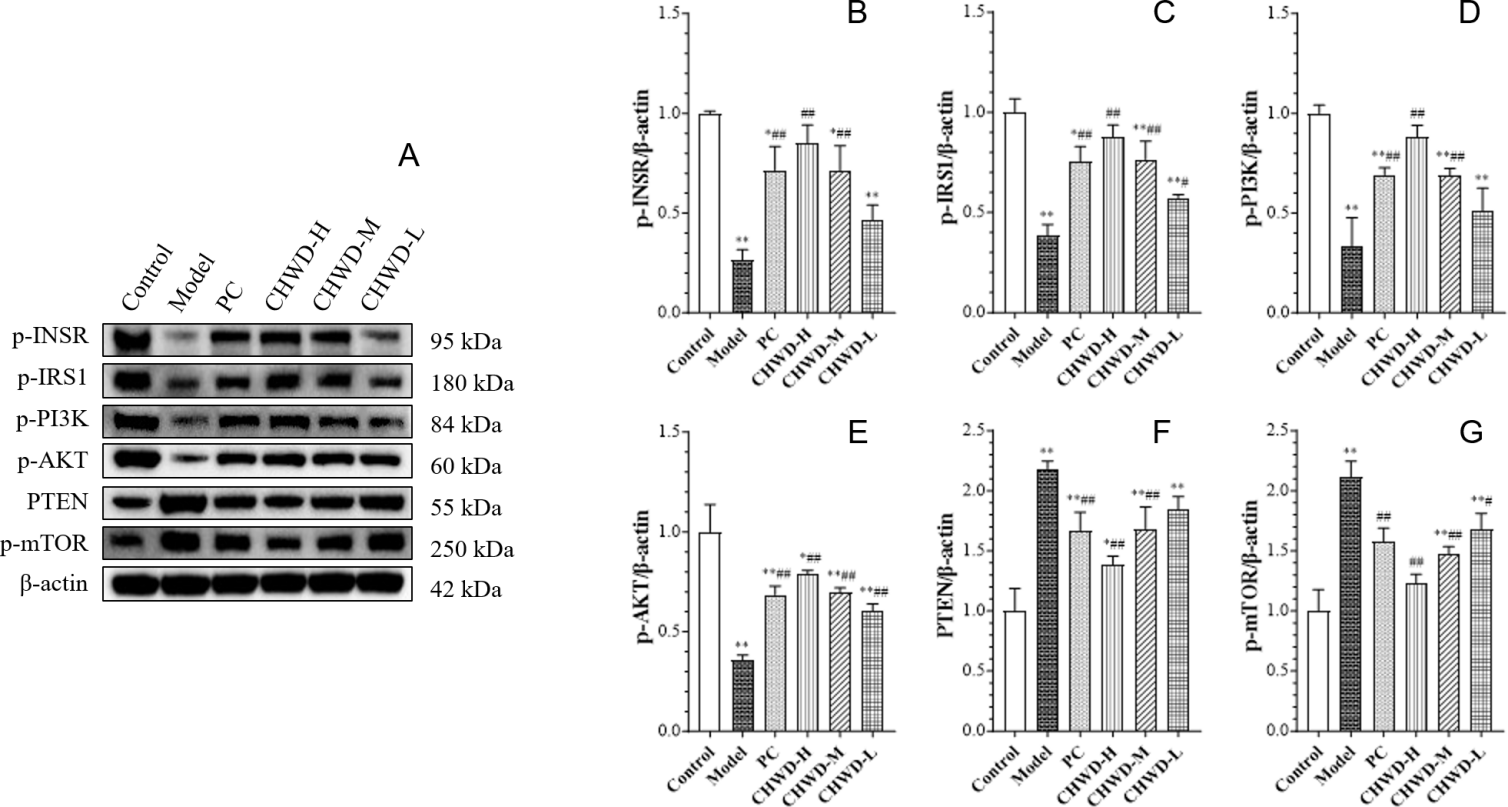
CHWD regulates the PTEN/PI3K/AKT signaling pathway in omental adipose tissue of different groups of mice. Representative Western blot images **(A)** and quantitative analysis of phosphorylation levels and protein expression of **(B)** p-INSR, **(C)** p-IRS1, **(D)** p-PI3K, **(E)** p-AKT, **(F)** PTEN, and **(G)** p-mTOR. Data are presented as mean ± SD (n = 3). Statistical differences are based on *P* < 0.05 (*) and *P* < 0.01 (**) vs. Control group and based on *P* < 0.05 (#) and *P* < 0.01 (##) vs. Model group.

To elucidate the molecular mechanism underlying the metabolic benefits of CHWD, the expression of proteins involved in insulin signaling cascade was investigated. The expression of PTEN, a negative regulator of the PI3K/AKT pathway, was significantly upregulated in the model group but significantly suppressed by CHWD treatment (High and Medium doses) (**Figure 4**).

Consistent with PTEN inhibition, CHWD treatment robustly reactivated the insulin signaling pathway. The phosphorylation levels of the insulin receptor (p-INSR), insulin receptor substrate-1 (p-IRS1), and PI3K (p-PI3K) were significantly increased in the CHWD-H and CHWD-M groups compared to the model group. Consequently, the phosphorylation of AKT (p-AKT), the central effector of insulin action, was significantly upregulated in all CHWD-treated groups (**Figure 4**).

**Figure 5:**
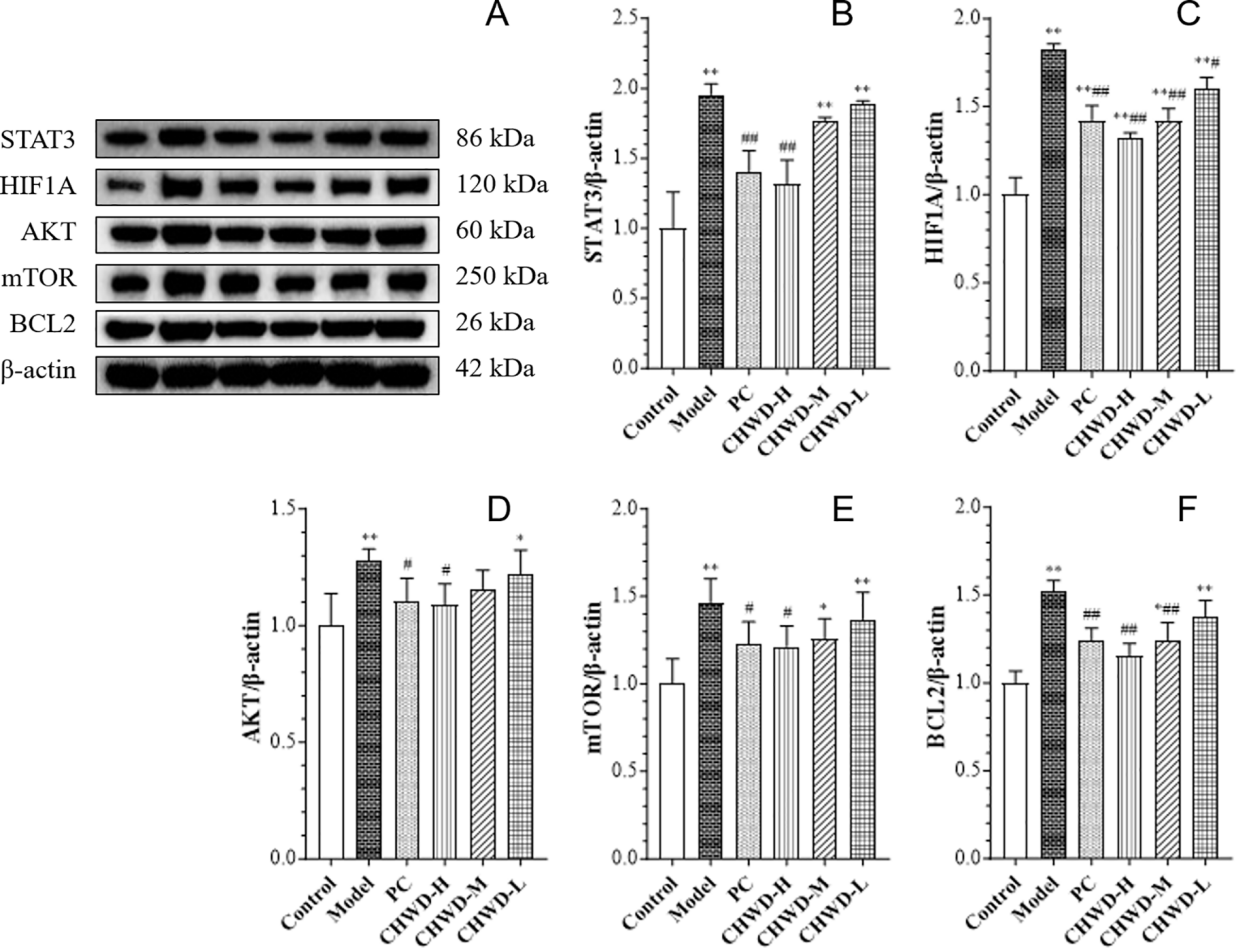
Effect of CHWD on protein expression of STAT3, BCL2, HIF1A, AKT, and mTOR in omental tissues of different groups of mice. Representative Western blots **(A)** and densitometric analysis of **(B)** STAT3, **(C)** HIF1A, **(D)** AKT, **(E)** mTOR, and **(F)** BCL2. Data are expressed as mean ± SD (n = 3). Statistical differences are based on *P* < 0.05 (*) and *P* < 0.01 (**) vs. Control group and based on *P* < 0.05 (#) and *P* < 0.01 (##) vs. Model group.

Interestingly, despite the activation of AKT, the phosphorylation of its downstream target mTOR (p-mTOR) was significantly downregulated by CHWD (**Figure 5**). This was accompanied by reduced total mTOR and AKT expression, which may reflect adaptive downregulation of pathway metabolites (**Figure 4**). This distinctive “high p-AKT/low p-mTOR” profile suggests that CHWD exerts a molecular brake on lipogenesis by suppressing mTOR signaling.

### 3.5 CHWD remodels the omental immune microenvironment by suppressing hypoxia and modulating macrophage polarization

Given the typical association between HFD-induced obesity is typically associated with tissue hypoxia and oxidative stress, we further investigated whether CHWD could remodel the pathological microenvironment of omental adipose tissue. HFD-induced obesity is typically associated with tissue hypoxia and oxidative stress. The results showed that the expression of HIF1A (a marker of hypoxia), STAT3 (a key inflammatory transcription factor), and BCL2 was significantly elevated in the model group, whereas CHWD treatment significantly downregulated these markers, indicating an improvement in tissue oxygenation and a reduction in inflammatory stress (**Figure 5**).

**Figure 6:**
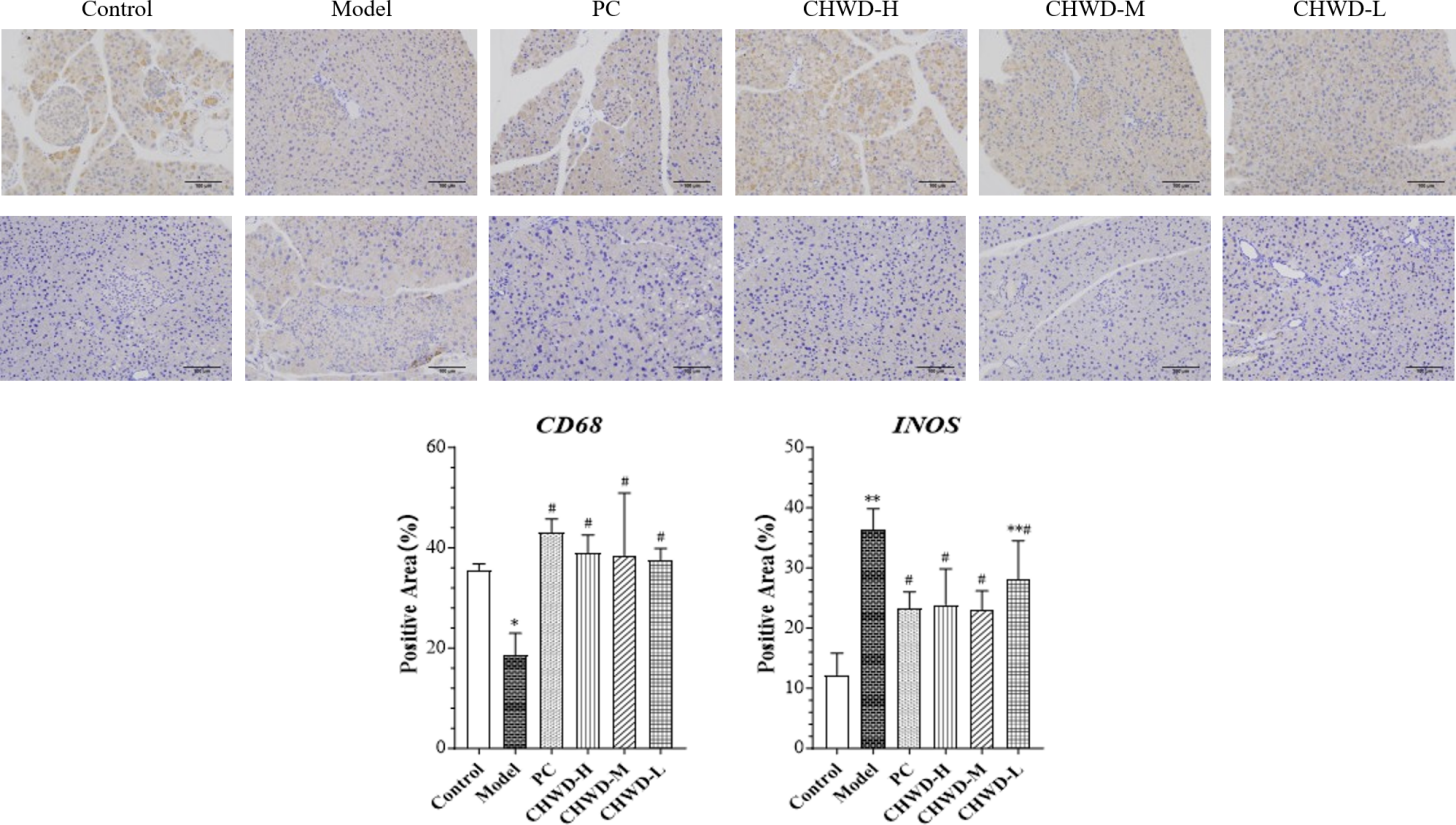
Immunohistochemical analysis of macrophage polarization markers (CD68 and iNOS) in omental tissue. Representative images of CD68 and iNOS staining (magnification, ×200) and quantitative analysis of the positive area. Data are expressed as mean ± SD (n = 3). Statistical differences are based on * P < 0.05 (*), and** P < 0.01 (**) vs. Control group and based on; # P < 0.05 (#) and, ## P < 0.01 (##) vs. Model group.

Immunohistochemical analysis revealed a distinct phenotypic reconfiguration of omental macrophages. Interestingly, compared to the Normal Control group, the model group exhibited a significant reduction in CD68 expression, suggesting altered macrophage distribution, or phenotypic marker expression pool, or immune dysregulation under chronic lipid overload. CHWD intervention effectively reversed this decline; CD68 expression was significantly restored in the High-, Medium-, and Low-dose groups compared to the model group (**Figure 6**). Crucially, this restoration of macrophage abundance was not accompanied by pro-inflammatory activity. Instead, the expression of iNOS, which was elevated in the model group, was significantly suppressed by CHWD treatment (**Figure 6**).

Collectively, these data indicated that CHWD remodeled the immune microenvironment through a “restoration-functional restraint” strategy, whereby macrophage abundance is restored (high CD68 expression) to support tissue homeostasis, while their cytotoxic effector phenotype is concomitantly restrained (low iNOS expression).

## 4 Discussion

This study demonstrates that CHWD exerts a systemic protective effect against HFD-induced obesity by orchestrating a functional signaling uncoupling and remodeling the liver-omentum axis. Rather than acting on a single isolated target, CHWD functions as a multi-target modulator that recalibrates the PTEN/AKT/mTOR cascade (22), which in turn dictates both metabolic flux and the immune microenvironment of visceral adipose tissue. By transitioning from a HFD to standard chow during intervention, we effectively mimicked the clinical reality of combined dietary management and pharmacological therapy, allowing for a more rigorous evaluation of CHWD’s potential to re-establish metabolic equilibrium.

The successful establishment of the HFD-induced obesity model (**Figure 1**) and the subsequent alleviation of hepatic steatosis by CHWD (**Figure 2**) highlight the systemic efficacy of this classical formula. A critical discovery in our study is that CHWD’s metabolic benefits are mediated through the liver-omentum axis. In the obese state, the greater omentum transitions into a pro-inflammatory reservoir, draining toxic mediators directly into the liver via the portal vein (23). Our results show that CHWD restores circulating Omentin-1 levels while suppressing systemic endotoxemia (**Figure 3**). This restoration of Omentin-1 is not merely a marker of improvement but a functional prerequisite for the subsequent sensitization of insulin signaling pathways (24, 25), serving as a vital endocrine bridge between omental health and hepatic metabolic stability (26).

To address the molecular “how” and “why” of CHWD’s action, we focused on the PTEN/PI3K/AKT/mTOR signaling cascade (**Figures 4**, **5**). Traditionally, PTEN acts as a “brake” on insulin signaling (27); its overexpression in obesity leads to insulin resistance. We found that CHWD robustly suppresses PTEN expression, thereby releasing this molecular brake and reactivating the upstream insulin cascade (INSR/IRS1/PI3K), as evidenced by the significant increase in p-AKT levels (**Figure 4**).

However, a pivotal “mechanistic uncoupling” was observed: despite the activation of AKT, the downstream phosphorylation of mTOR was paradoxically suppressed by CHWD (**Figure 5**). This “high p-AKT/low p-mTOR” profile is the core of our mechanistic narrative. In standard insulin resistance, compensatory hyperinsulinemia often drives mTOR-mediated lipogenesis even when glucose disposal is impaired (28). By functionally uncoupling AKT from mTOR, CHWD selectively promotes glucose uptake (via AKT) while simultaneously applying a molecular brake on mTOR-driven lipid synthesis (29). This unique dual-regulatory mechanism explains why CHWD can alleviate hepatic steatosis and dyslipidemia without the side effects associated with non-selective insulin sensitizers. The core of our mechanistic narrative is the ‘high p-AKT/low p-mTOR’ profile. By uncoupling AKT from mTOR, CHWD selectively promotes glucose disposal (via AKT) while concurrently applying a molecular brake on mTOR-driven lipid synthesis. This dual-regulation avoids the side effects of non-selective insulin sensitizers, such as weight gain.

The recalibration of intracellular phosphorylation extends beyond metabolic flux to the remodeling of the omental immune microenvironment. The PTEN/AKT/mTOR axis is a well-known rheostat for macrophage polarization. Our data reveal that HFD triggers severe tissue hypoxia and inflammatory stress (30), indicated by elevated HIF1A, STAT3, and BCL2 (**Figure 5**). Through the suppression of PTEN and the subsequent signaling uncoupling, CHWD shifts the functional state of omental macrophages. Rather than inducing simple immune cell depletion, CHWD employs a “restoration-functional restraint” strategy. Immunohistochemical analysis (**Figure 6**) confirms that while the CD68^+^ macrophage population is maintained to support tissue homeostasis, their cytotoxic effector functions - marked by iNOS expression - are strictly restrained (31, 32). This phenotypic shift from a pro-inflammatory to a non-cytotoxic state effectively reduces the inflammatory “load” delivered to the liver (33), further stabilizing the liver-omentum axis. Current data reveal a reversal of the pro-inflammatory phenotype, but future research will utilize flow cytometry or single-cell sequencing to further subdivide macrophage subsets.

## 5 Conclusion

In summary, CHWD ameliorates obesity through coordinated regulation of metabolic signaling and inflammatory microenvironments. By targeting PTEN, CHWD restores insulin signaling via AKT activation, suppresses mTOR-dependent lipogenesis, normalizes Omentin-1 secretion, and re-educates macrophages toward a non-cytotoxic phenotype. These findings provide mechanistic support for the clinical use of CHWD in metabolic syndrome and identify the PTEN/AKT/mTOR axis as a promising therapeutic node. Future studies employing PTEN- or Omentin-1–deficient models, as well as component-level biochemical interaction analyses, are warranted to establish causal relationships and identify the active constituents responsible for these effects (34).

## Data Availability

The datasets presented in this study can be found in online repositories. The names of the repository/repositories and accession number(s) can be found in the article/**Supplementary Material**.
