# Supplementary figures and images for: Chaihu-Wendan Decoction alleviates obesity through PTEN-mediated uncoupling of metabolic signaling and macrophage activation
Source: Front Endocrinol (Lausanne). 2026 Apr 15;17:1779657. doi: 10.3389/fendo.2026.1779657 (PMC13124501; doi:10.3389/fendo.2026.1779657)

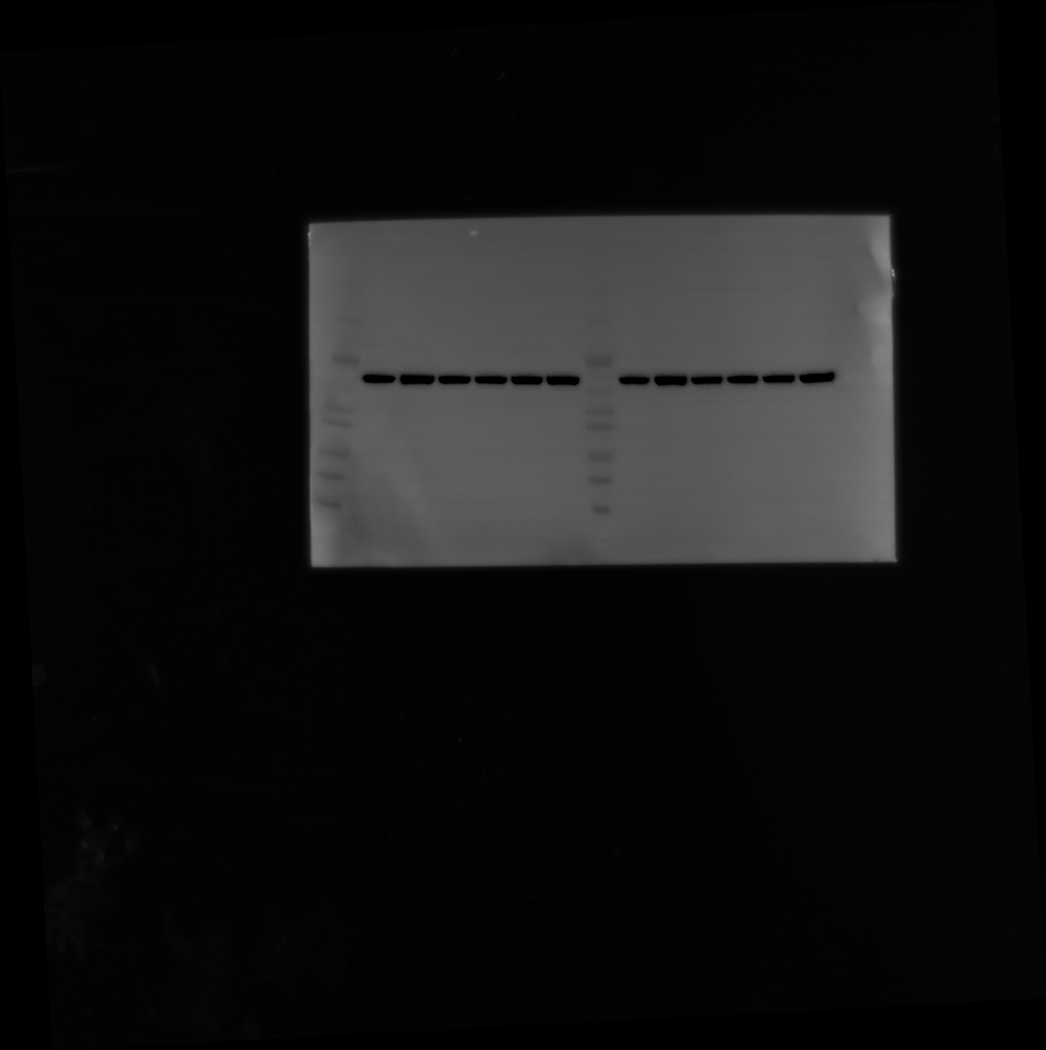

Supplement: Supplementary file 1 [file Image1.tiff]

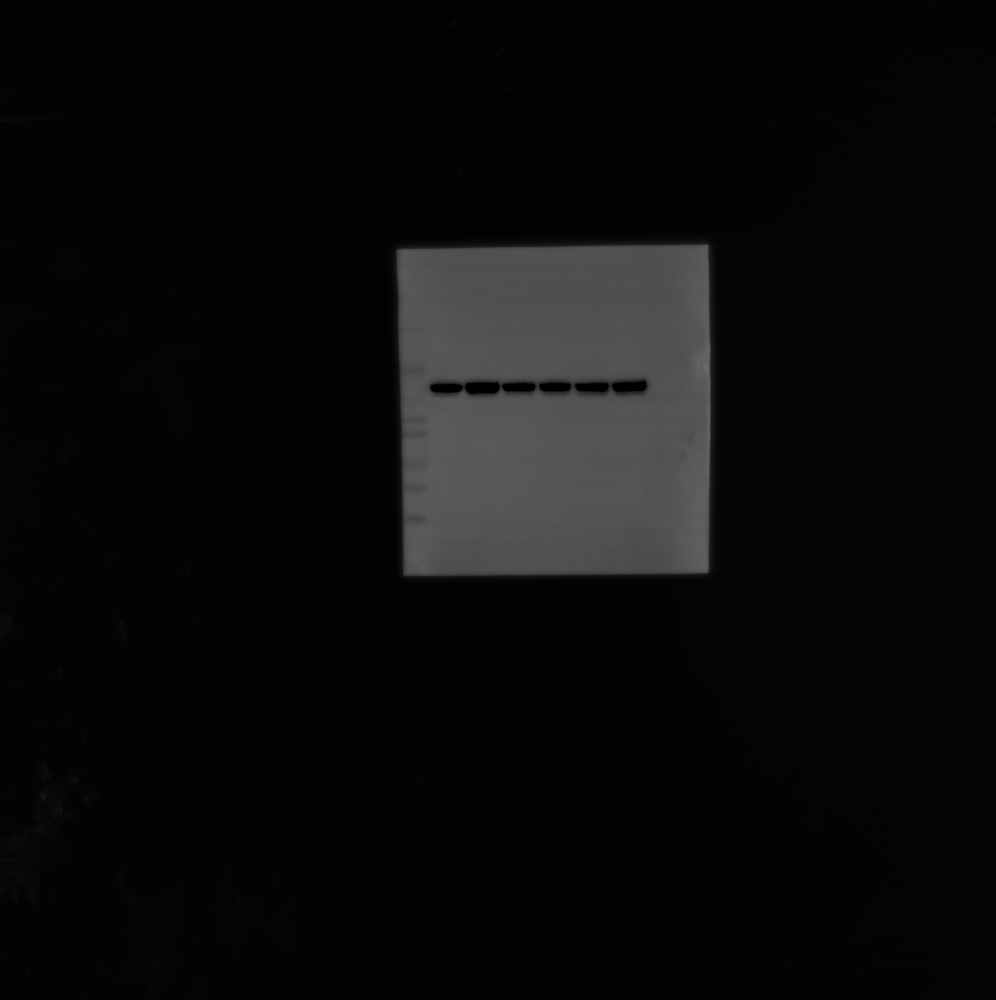

Supplement: Supplementary file 2 [file Image2.tiff]

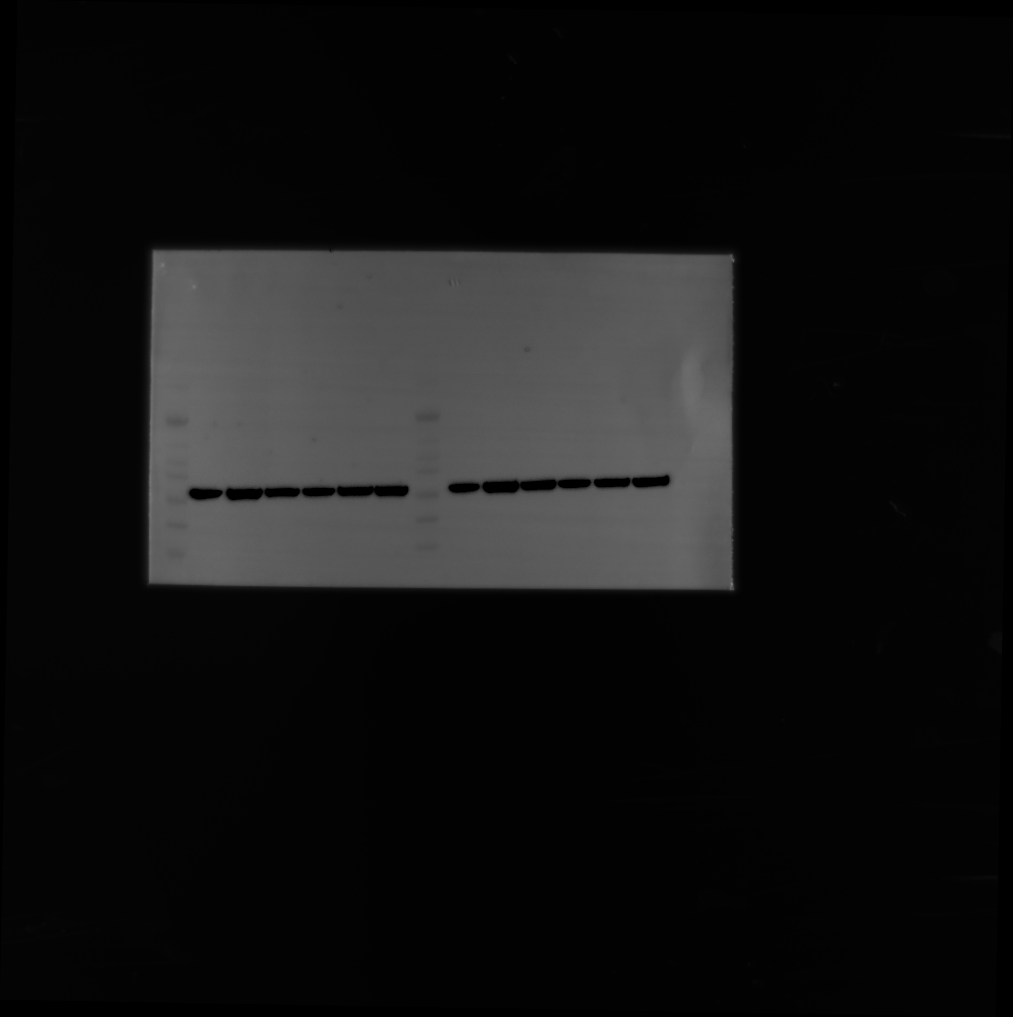

Supplement: Supplementary file 3 [file Image3.tiff]

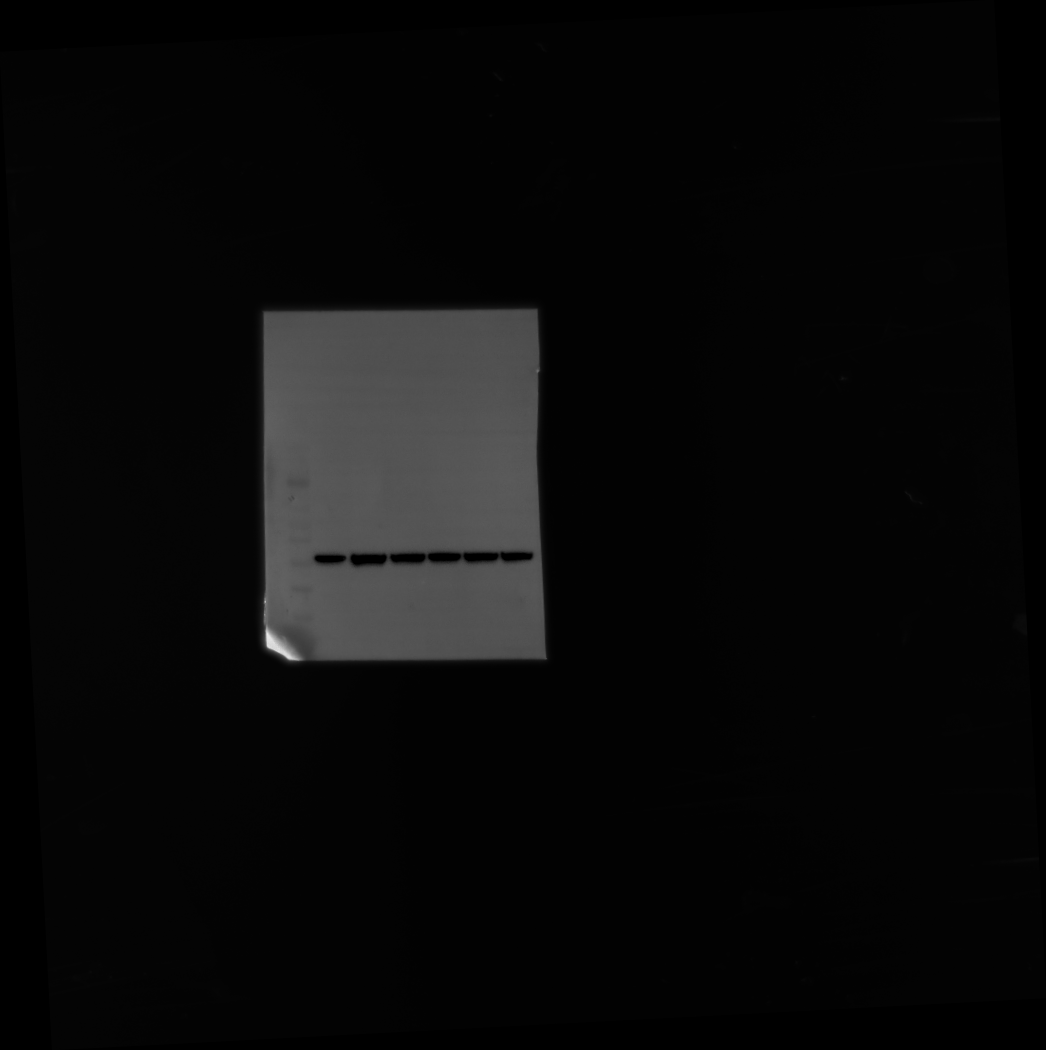

Supplement: Supplementary file 4 [file Image4.tiff]

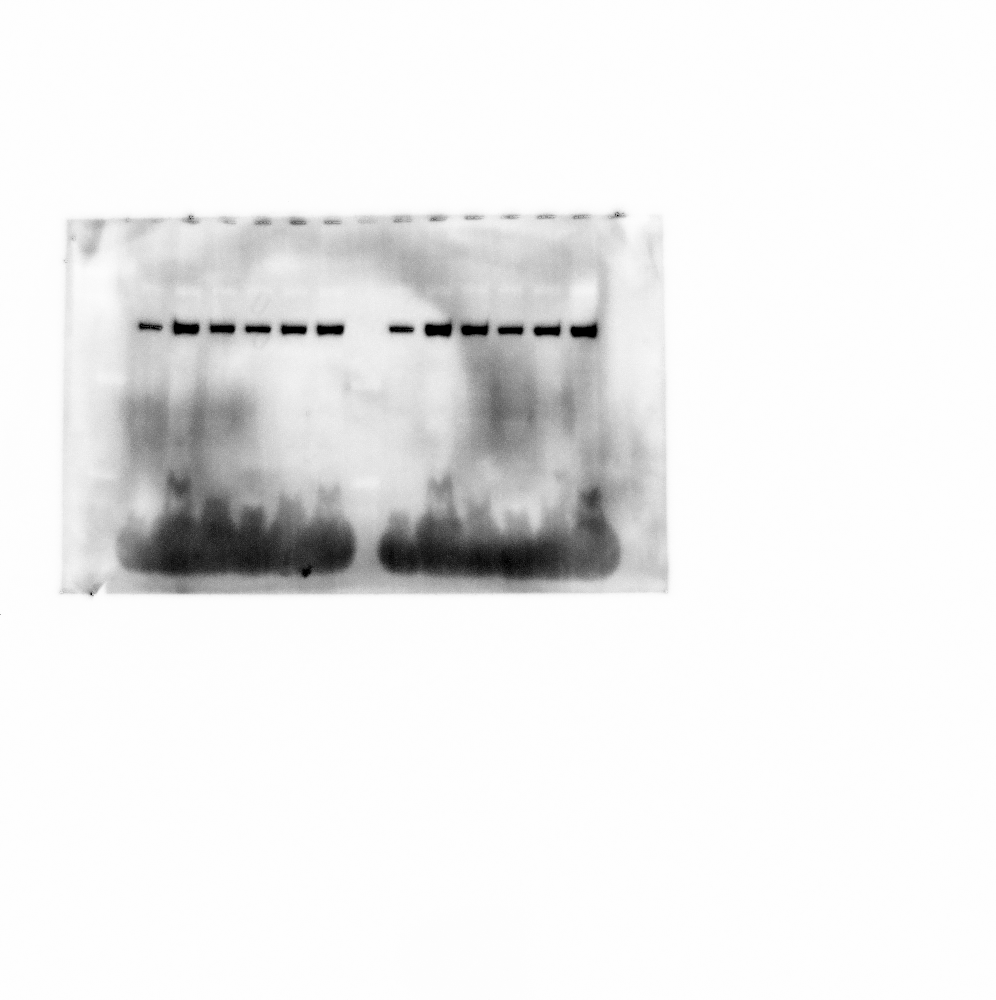

Supplement: Supplementary file 5 [file Image5.tiff]

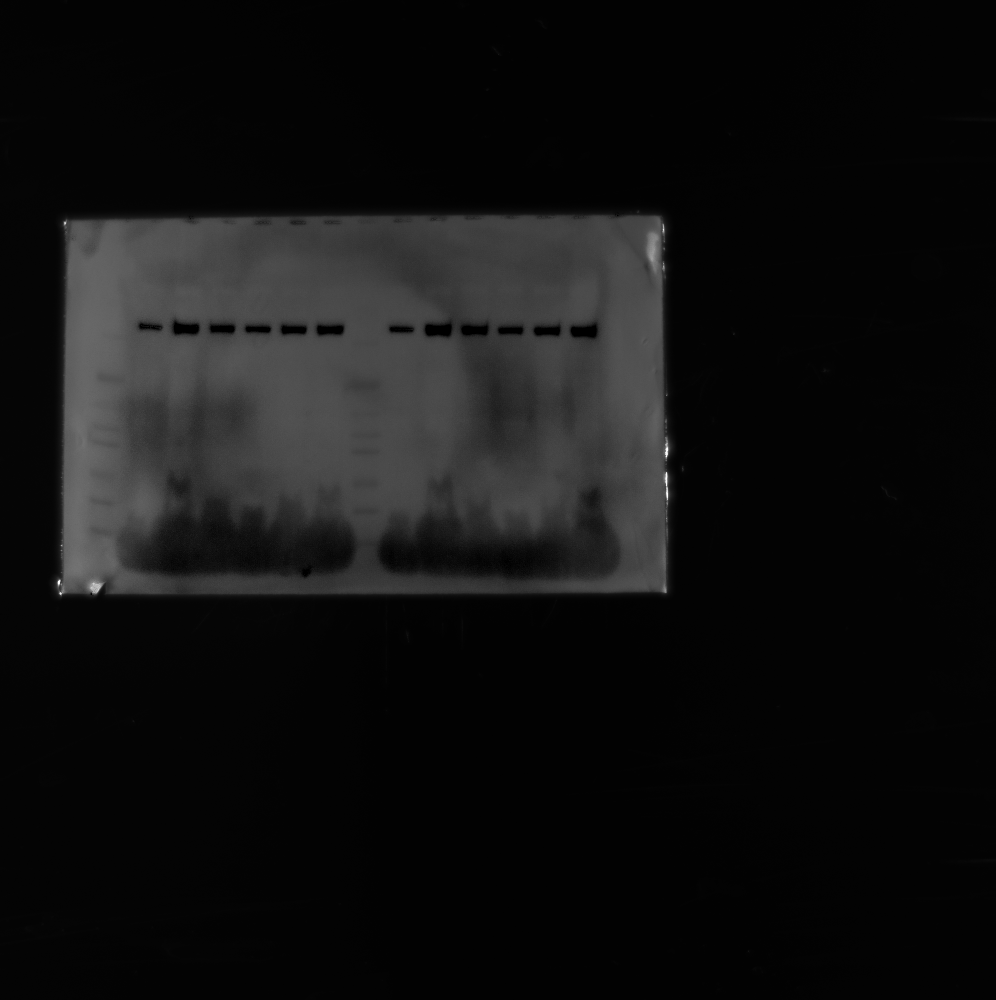

Supplement: Supplementary file 6 [file Image6.tiff]

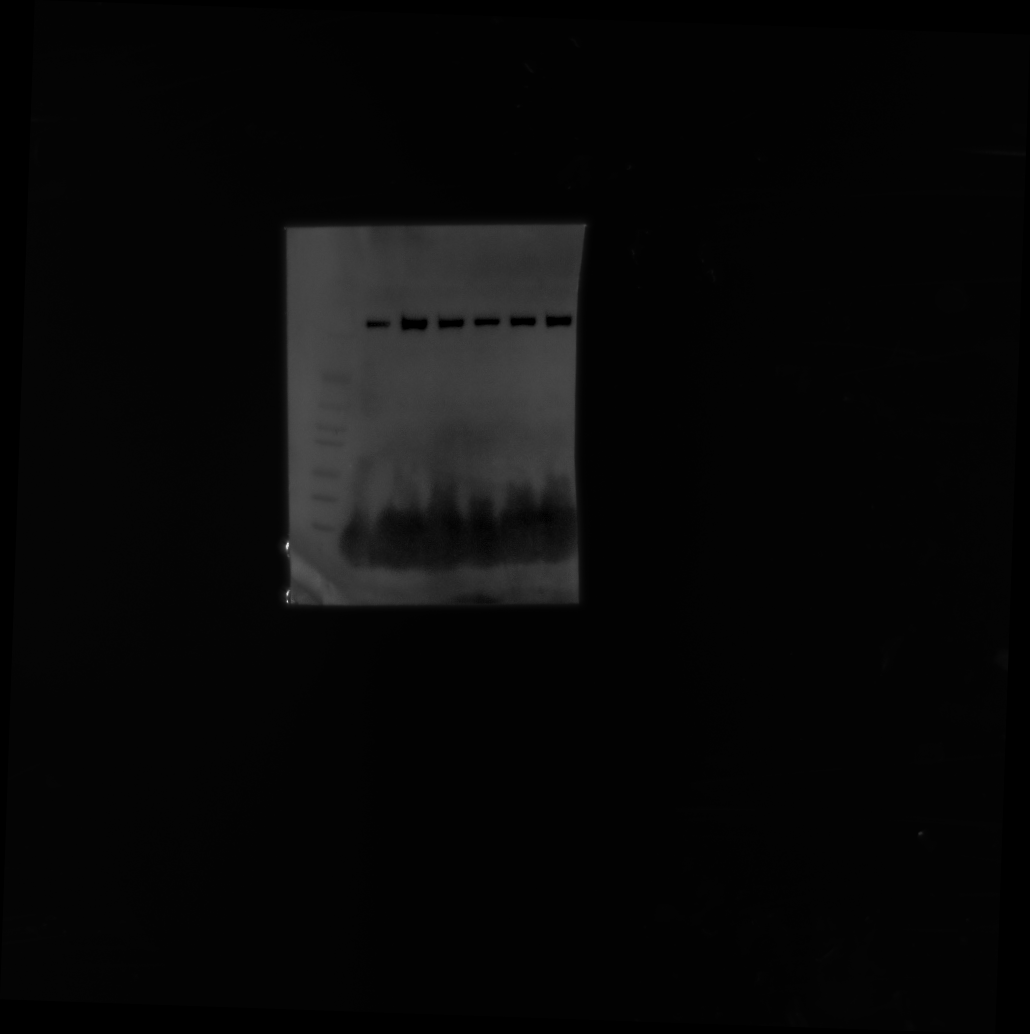

Supplement: Supplementary file 7 [file Image7.tiff]

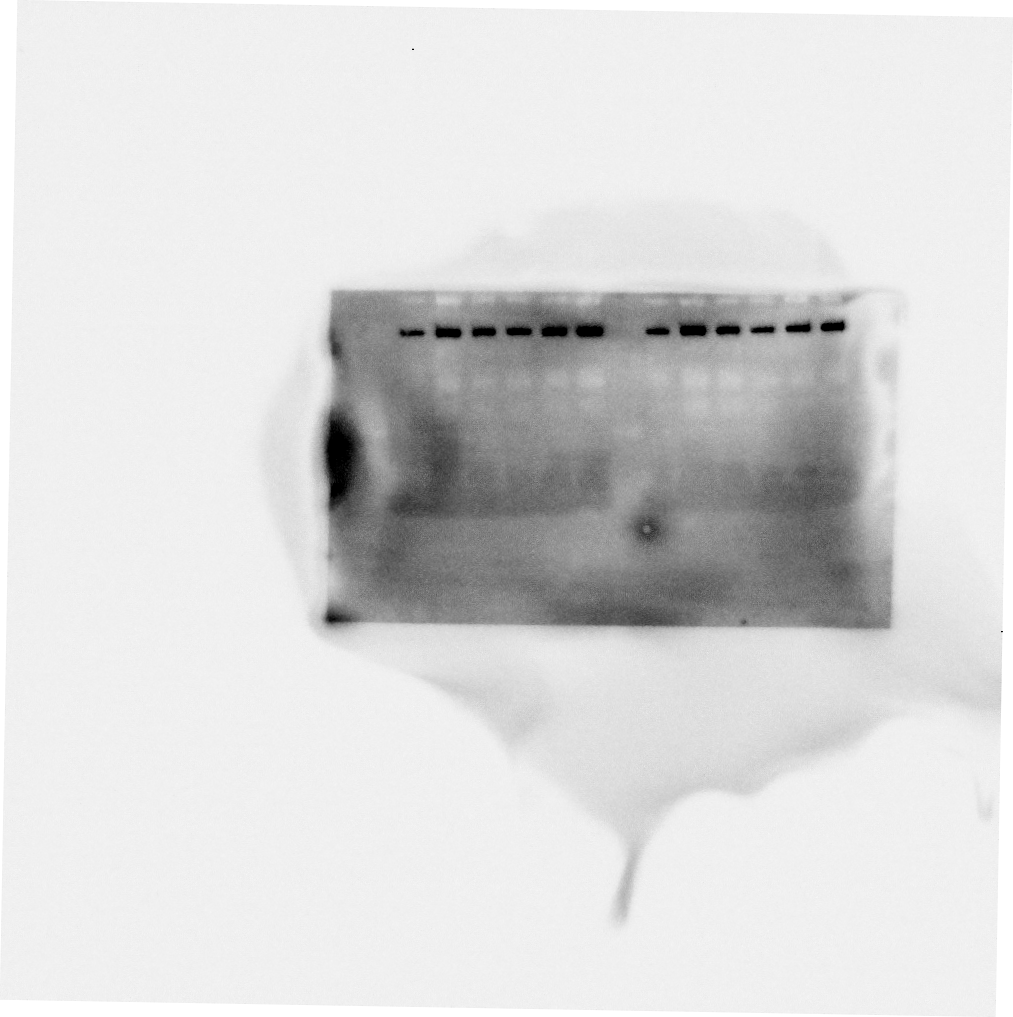

Supplement: Supplementary file 8 [file Image8.tiff]

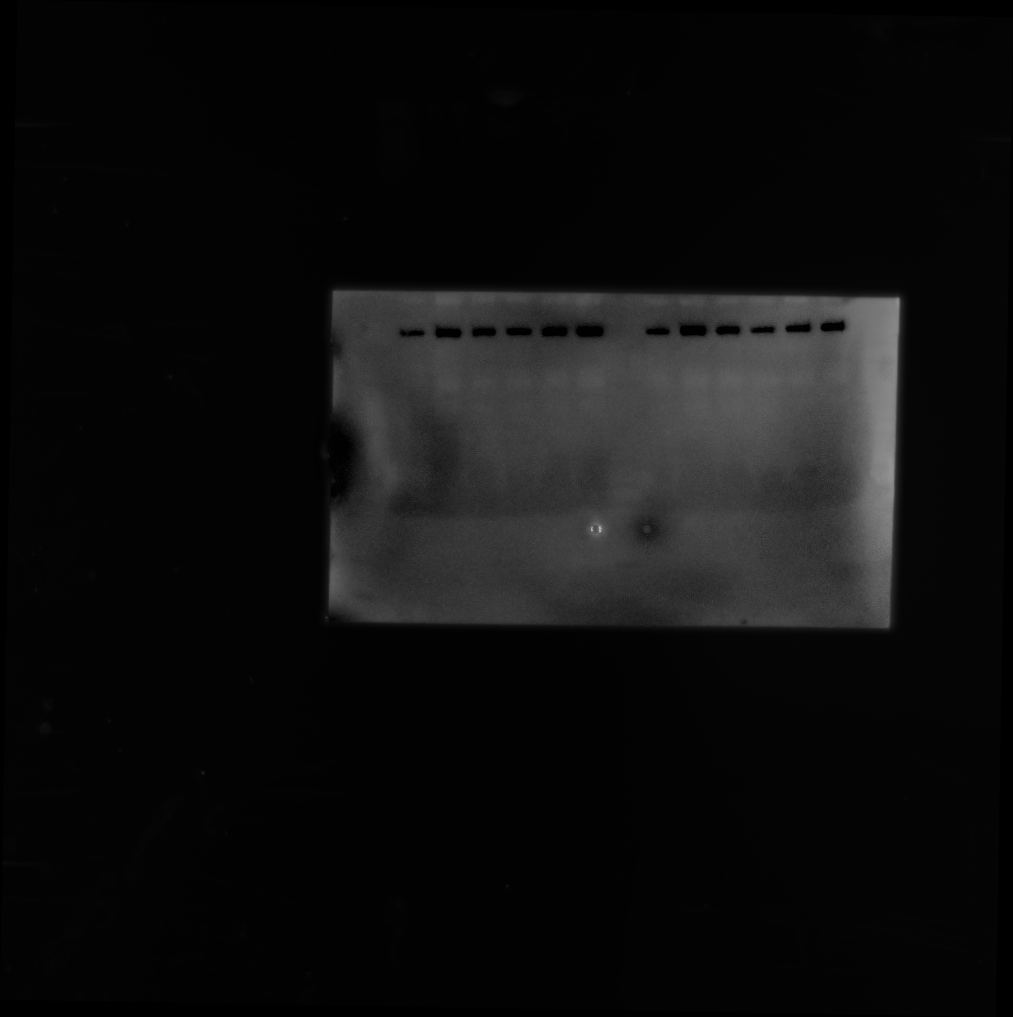

Supplement: Supplementary file 9 [file Image9.tiff]

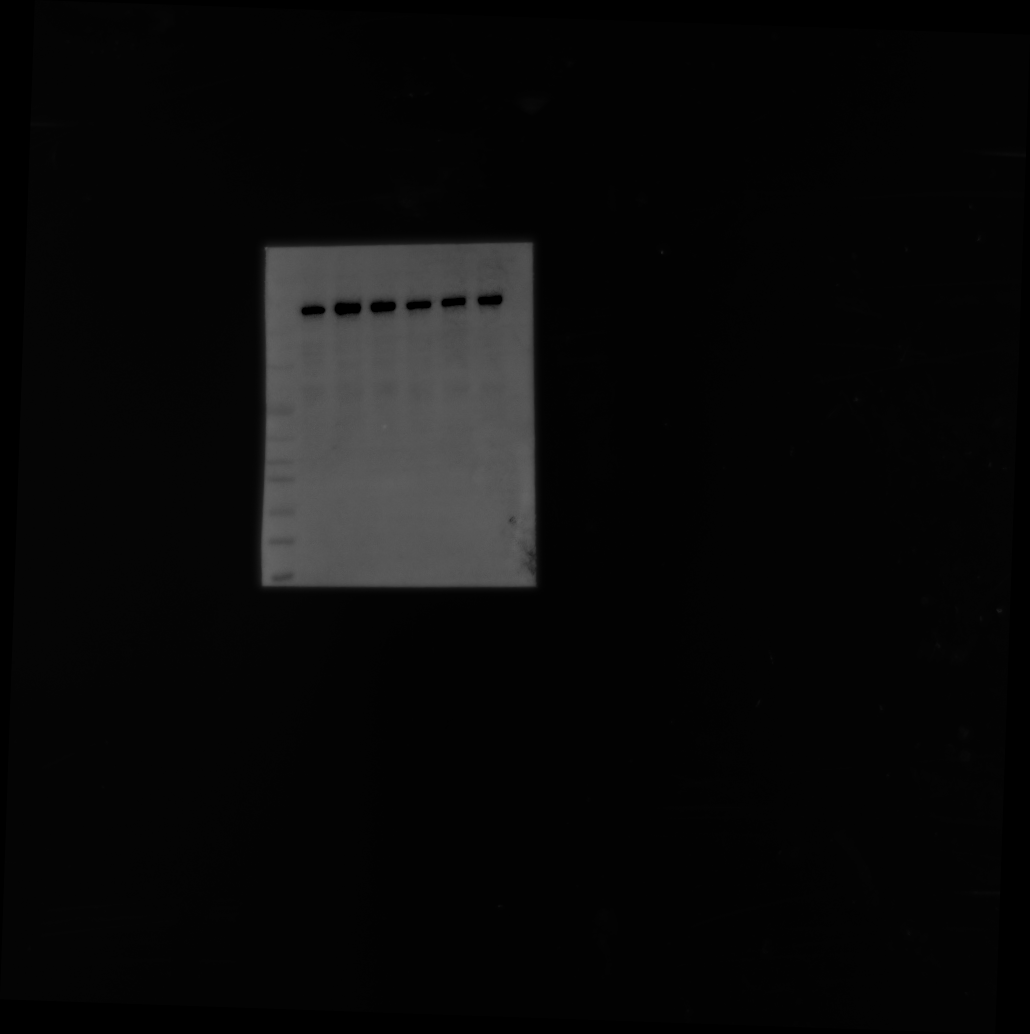

Supplement: Supplementary file 10 [file Image10.tiff]

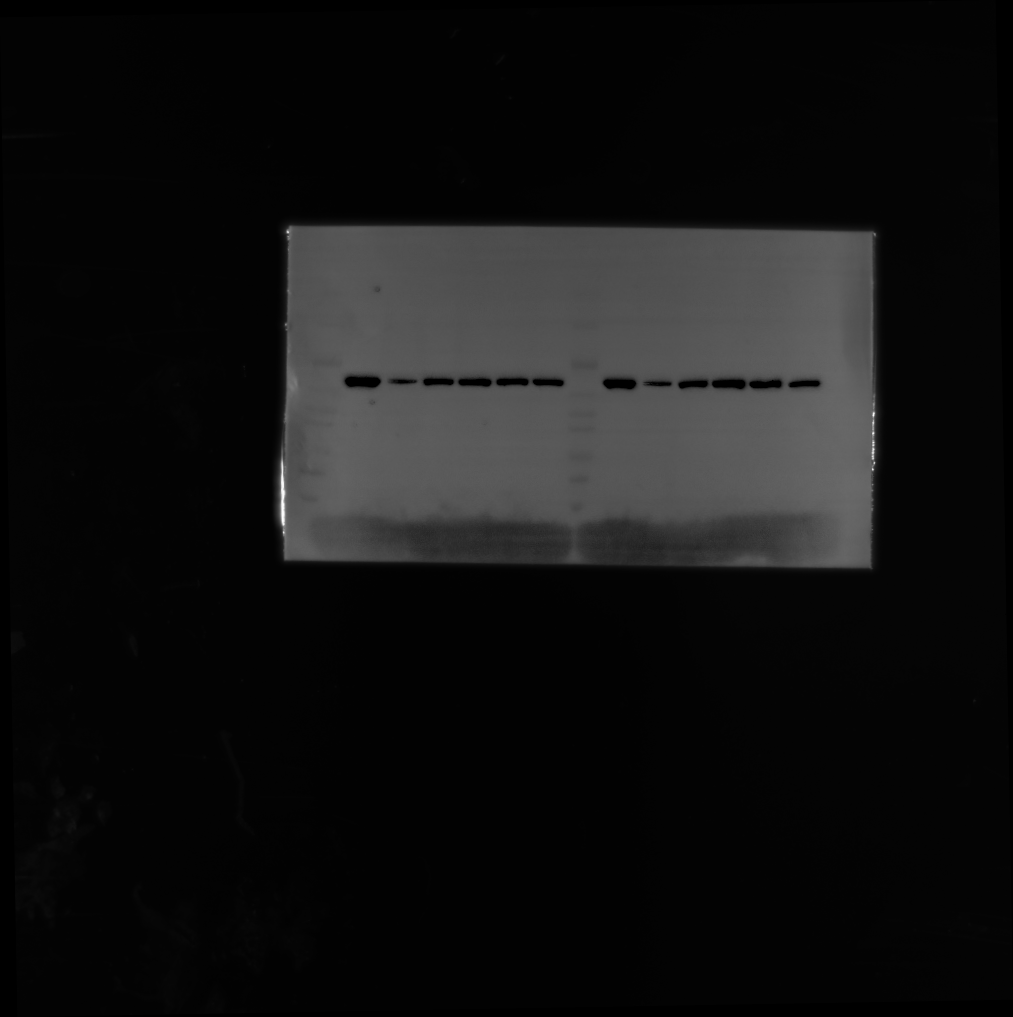

Supplement: Supplementary file 11 [file Image11.tiff]

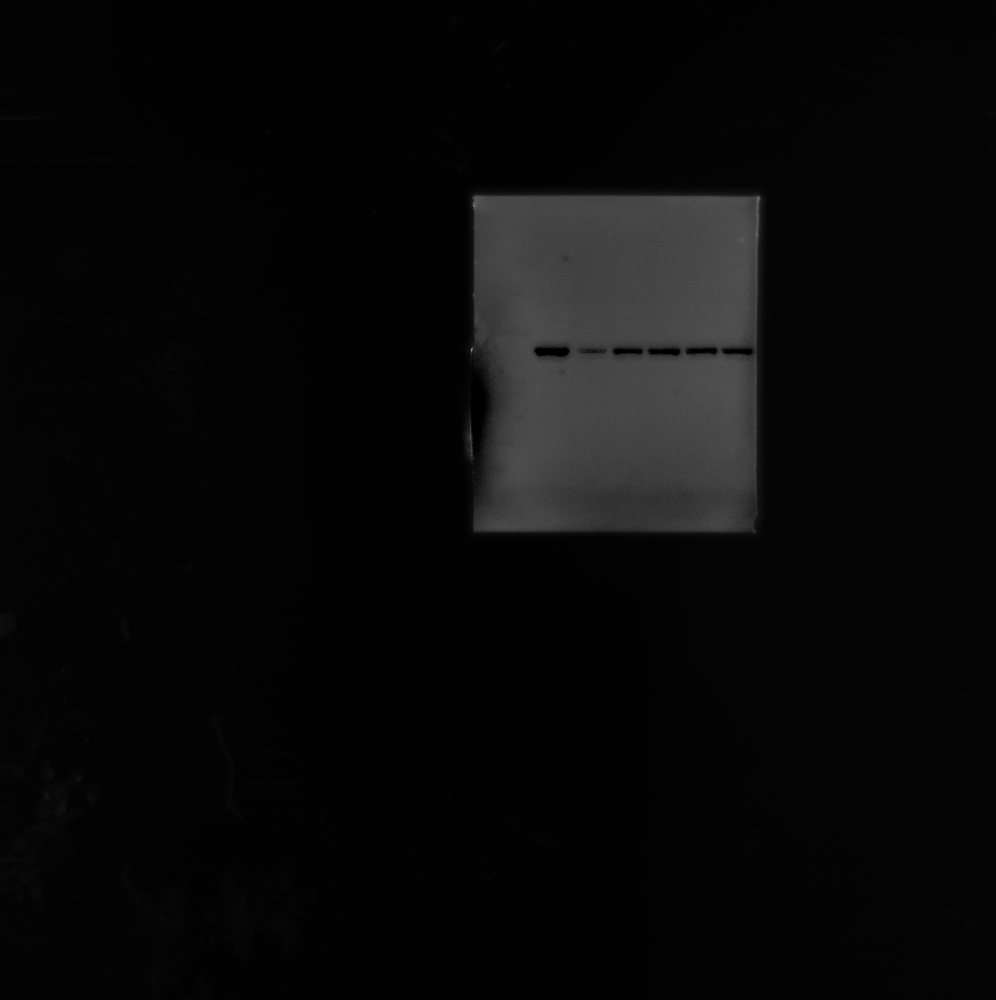

Supplement: Supplementary file 12 [file Image12.tiff]

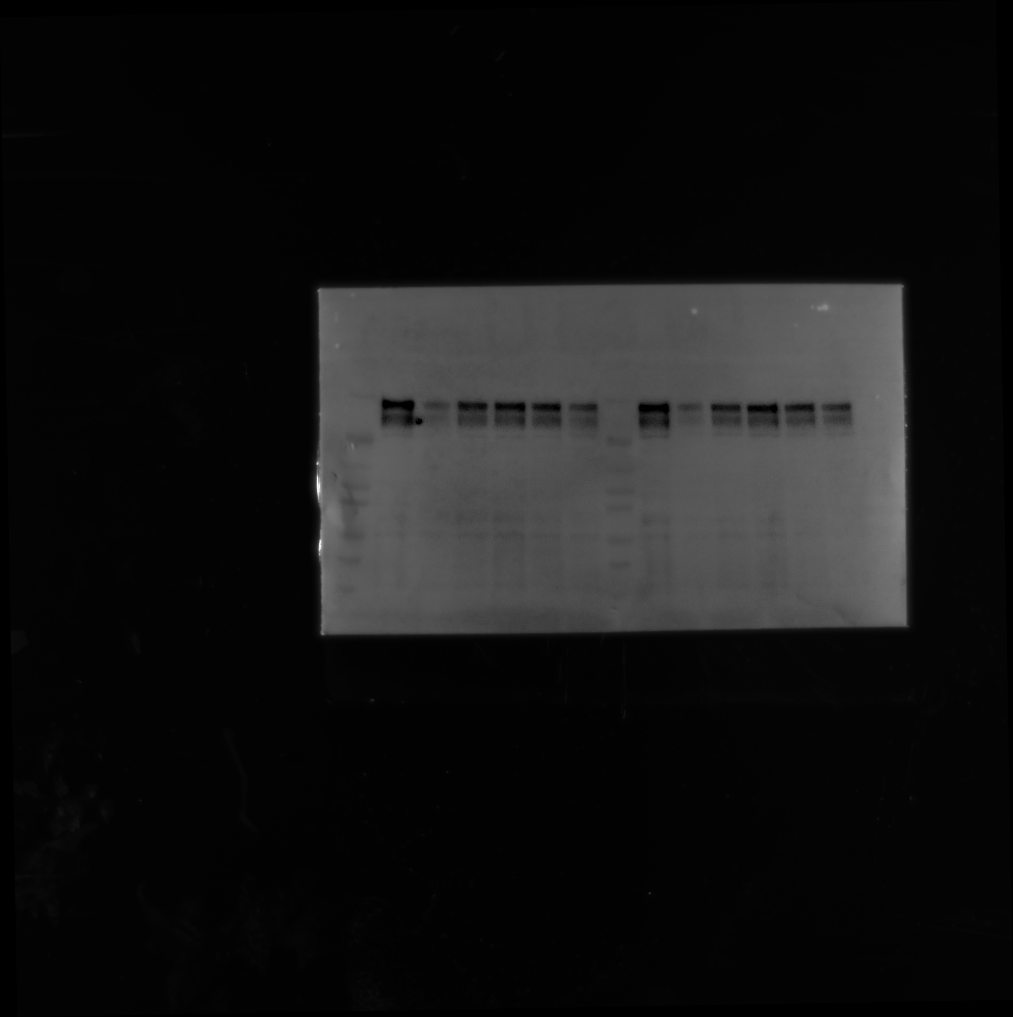

Supplement: Supplementary file 13 [file Image13.tiff]

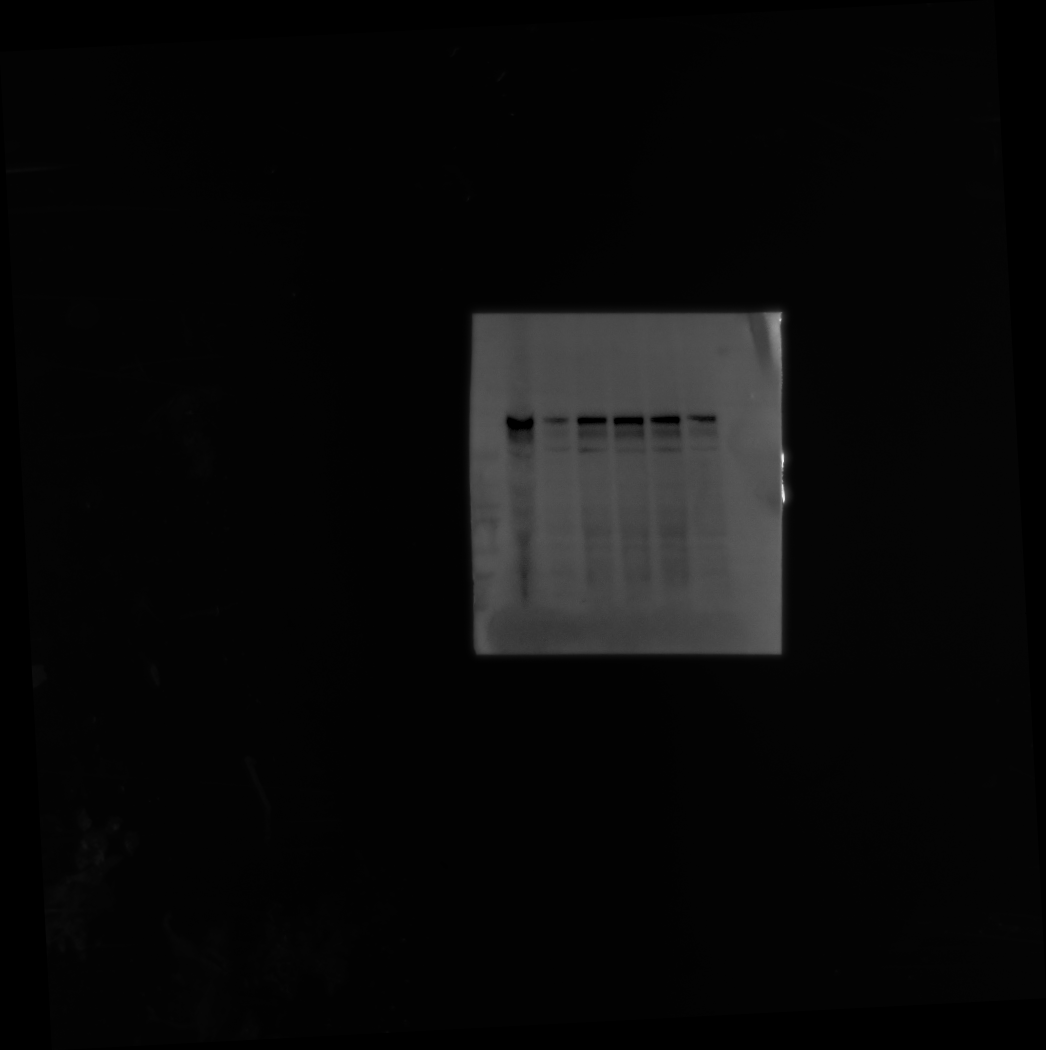

Supplement: Supplementary file 14 [file Image14.tiff]

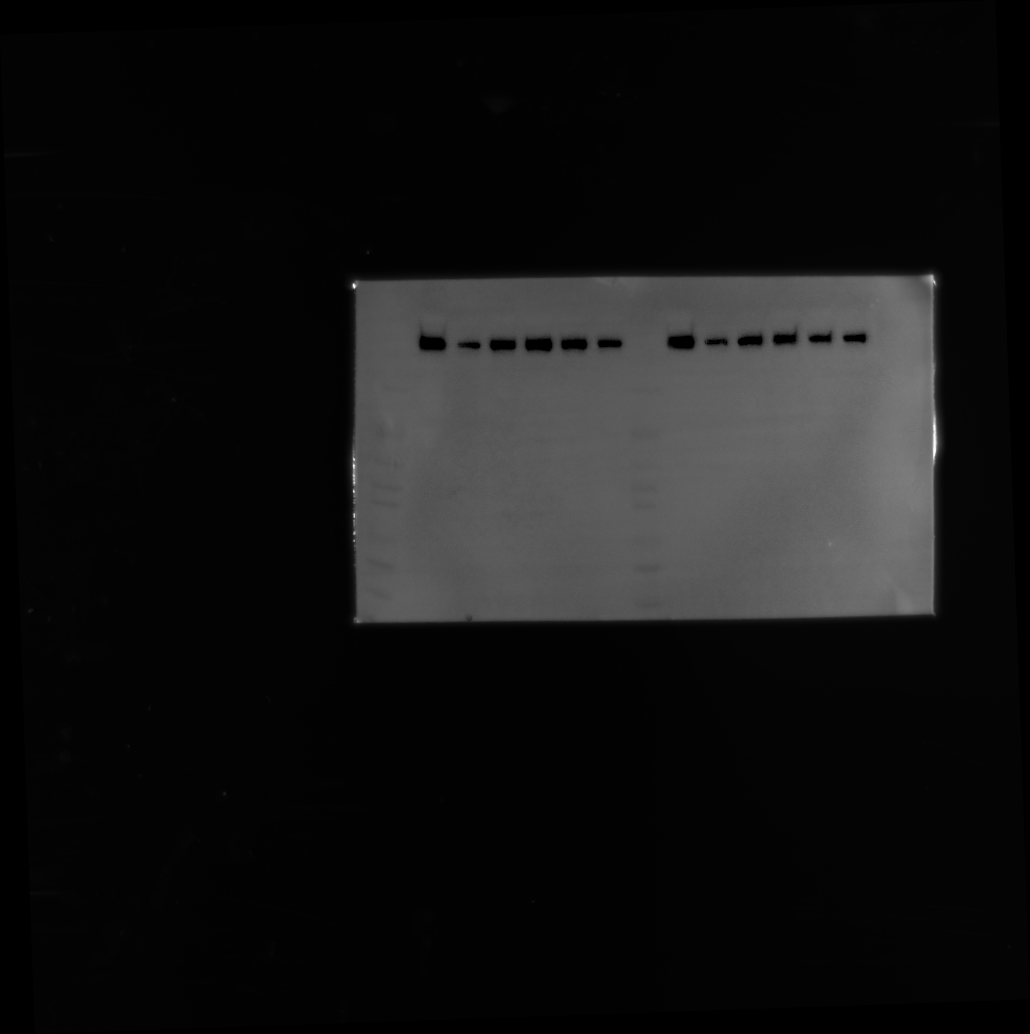

Supplement: Supplementary file 15 [file Image15.tiff]
